# Perceived cognitive impairment in high school students in the United States

**DOI:** 10.3389/fpsyg.2022.1019159

**Published:** 2022-10-04

**Authors:** Grant L. Iverson, Ila A. Iverson

**Affiliations:** ^1^Department of Physical Medicine and Rehabilitation, Harvard Medical School, Boston, Massachusetts, United States; ^2^Department of Physical Medicine and Rehabilitation, Spaulding Rehabilitation Hospital, Charlestown, Massachusetts, United States; ^3^Department of Physical Medicine and Rehabilitation, Spaulding Research Institute, Charlestown, Massachusetts, United States; ^4^MassGeneral Hospital for Children Sports Concussion Program, Boston, Massachusetts, United States; ^5^Department of Psychology, University of British Columbia, Vancouver, British Columbia, Canada

**Keywords:** cognitive impairment, depression, sleep, adolescents, adversity

## Abstract

**Introduction:**

Some youth experience cognitive difficulties that interfere with their ability to learn and function well in a school environment. We examined correlates of perceived cognitive impairment among high school students who completed a national survey conducted by the United States Centers for Disease Control and Prevention (CDC) in 2019.

**Methods:**

Participants were high school students (grades 9–12) who completed the Youth Risk Behavior Survey (YRBS) in 2019. The CDC uses this survey to monitor risk behaviors. Students answered the following question ‘Because of a physical, mental, or emotional problem, do you have serious difficulty concentrating, remembering, or making decisions?’ as either ‘yes’ or ‘no.’ Student responses to this question were analyzed in relation to demographic variables and variables pertaining to adversity, mental health problems, and drug use.

**Results:**

The sample included 8,349 students between the ages of 14 and 18, with 4,093 boys (49%) and 4,256 girls (51%). A large proportion reported having serious difficulty concentrating, remembering, or making decisions due to physical, mental, or emotional problems (38%). A significantly larger proportion of girls (45%) than boys (30%) reported experiencing cognitive impairment [*χ*^2^(1) = 212.23, *p* < 0.001; Odds Ratio = 1.95, 95% confidence interval = 1.78–2.13]. Youth who exercised regularly were significantly less likely to report cognitive impairment. Binary logistic regression was used to examine the associations between perceived cognitive impairment and adversity, mental health, and lifestyle variables separately for boys [*χ*^2^(11) = 569.158, *p* < 0.001; Nagelkerke *R*^2^ = 0.212] and girls [*χ*^2^(11) = 1,026.189, *p* < 0.001; Nagelkerke *R*^2^ = 0.321]. Being bullied, feeling unsafe or threatened at school, getting very low grades, insufficient sleep, and using illicit drugs were independently associated with perceived cognitive impairment in both boys and girls—after controlling for associations with depression and suicidality. Youth who denied mental health problems, psychosocial adversities, and using illicit drugs reported much lower rates of perceived cognitive impairment (boys = 13%, girls = 15%).

**Conclusion:**

A remarkably large proportion of high school students in the United States reported experiencing serious difficulty with their cognitive functioning over the past year. Girls were significantly more likely to endorse perceived cognitive difficulties compared to boys. There was a strong association between perceived cognitive impairment and the experience of psychosocial adversity.

## Introduction

Some adolescents experience significant cognitive difficulties in their daily lives. Those cognitive difficulties might be subjectively experienced, objectively measured using neuropsychological testing, or both. Youth with neurodevelopmental conditions, such as attention-deficit hyperactivity disorder (ADHD) (Pievsky and McGrath, 2018), mood disorders, such as depression (Allott et al., 2016) and bipolar disorder (Elias et al., 2017), medical problems, such as critical illnesses (Kachmar et al., 2018), pediatric heart surgery (Sterken et al., 2015), and sleep apnea (Krysta et al., 2017), and neurological problems, such as traumatic brain injuries (Gorgoraptis et al., 2019), brain tumors (de Ruiter et al., 2013), and epilepsy (Nickels et al., 2016) experience cognitive deficits as measured by neuropsychological tests. Moreover, subjectively experienced cognitive difficulties accompany many neurodevelopmental, psychiatric, and neurological disorders, and perceived cognitive difficulty is one of the diagnostic criteria for ADHD, major depressive disorder, and generalized anxiety disorder (American Psychiatric Association, 2013).

A question relating to perceived cognitive impairment is included in the Youth Risk Behavior Survey (YRBS), a national survey conducted by the United States Centers for Disease Control and Prevention (CDC). That question reads as follows: ‘Because of a physical, mental, or emotional problem, do you have serious difficulty concentrating, remembering, or making decisions?’ The Youth Risk Behavior Surveillance System, which includes both a nationally representative YRBS and separate state, local school district, territorial, and tribal surveys, is the largest youth public health surveillance system in the United States. The YRBS assesses a broad range of health risk behaviors, and it is subdivided into six categories: (i) behaviors that contribute to unintentional injury and violence; (ii) tobacco use; (iii) alcohol and other drug use; (iv) sexual behaviors that contribute to unintended pregnancy and sexually transmitted disease infection; (v) dietary behaviors; and (vi) physical inactivity (Underwood et al., 2020). The YRBS allows the CDC to monitor how risk behaviors fluctuate over time among high school students (grades 9–12), because it is administered every 2 years.

The purpose of this study is to examine perceived cognitive impairment in high school students who have completed the national YRBS. We hypothesized that students who reported experiencing adversity (e.g., sexual abuse, sexual assault, and bullying), mental health problems (e.g., depression and suicidality), or drug use would endorse cognitive impairment at greater rates than youth who did not report these psychosocial and mental health problems. Because insufficient sleep has been associated with greater physical, emotional, and cognitive symptoms in high school student athletes (McClure et al., 2014; Silverberg et al., 2016; Terry et al., 2021; Moran and Ingargiola, 2022), we hypothesized that insufficient sleep would be associated with greater endorsement of cognitive impairment. Given that researchers have reported that participation in team sports, and a high level of physical activity, are associated with better self-esteem and greater life satisfaction, and lower risk for psychological distress (Steptoe and Butler, 1996; Sabo et al., 2005; Brown et al., 2007; Taliaferro et al., 2008; Babiss and Gangwisch, 2009; Eime et al., 2013; Sibold et al., 2015; McMahon et al., 2017; He et al., 2018; Guddal et al., 2019), we hypothesized that these variables would be associated with lower rates of endorsing cognitive impairment.

## Materials and methods

### Survey methodology

The YRBS is a cross-sectional, school-based survey that is conducted every 2 years among students in grades 9–12 who attend both public and private schools in the United States. It has been done since 1991. The YRBS protects student privacy and allows for anonymous and voluntary participation by students. It is administered using a computer-scannable answer booklet during one class period (approximately 45 min). The protocol for the national YRBS has been reviewed and approved by the CDC’s Institutional Review Board, and the data for each survey, going back many years, are publicly available on their website.

In 2019, the school response rate was 75.1%, the student response rate was 80.3%, and the overall response rate (i.e., the product of the student response rate and the school response rate) was 60.3% [i.e., (student response rate) × (school response rate)] (Underwood et al., 2020). There were 13,872 questionnaires completed in 136 schools. Of these, 195 failed quality control and were excluded leaving 13,677 usable questionnaires. A questionnaire failed quality control if the student endorsed the same answer for 15 or more consecutive questions, or if fewer than 20 responses remained after editing.

### Survey questions and combined variables

The 2019 survey contained 99 questions with 89 of these included in the standard YRBS. Questions could be added or deleted at each different testing site, but it was required that at least 60 of the questions on the standard questionnaire remained. The question relating to perceived cognitive impairment was an additional question not included in the standard 89 questions (i.e., question #98). The cognitive impairment question was added to the YRBS for the first time in 2019. The survey questions, definitions of each variable, response options, and recall periods are available in the 2019 YRBS questionnaire and data user guide—found on the website.^1^

The focus of this study was student’s self-reported cognitive impairment. The 2019 YRBS included the question: ‘Because of a physical, mental, or emotional problem, do you have serious difficulty concentrating, remembering, or making decisions?’ The response options to this question were binary: ‘Yes’ or ‘No.’ The questions used in this study, and combined variables, are listed in Table 1.

**Table 1:** Survey questions and combined variables.

1. Felt Unsafe at School: QN15. During the past 30 days, on how many days did you not go to school because you felt you would be unsafe at school or on your way to or from school?
2. Threatened on School Property: QN16. During the past 12 months, how many times has someone threatened or injured you with a weapon such as a gun, knife, or club on school property?
3. *Felt Unsafe or Threatened at School: Responded affirmatively to Q15 or Q16.
4. *Lifetime Sexual Abuse/Assault: Q19. Have you ever been physically forced to have sexual intercourse when you did not want to?
5. Sexual Assault/Abuse Past 12 Months: QN20. During the past 12 months, how many times did anyone force you to do sexual things that you did not want to do? (Count such things as kissing, touching, or being physically forced to have sexual intercourse.)
6. Dating Violence: QN22. During the past 12 months, how many times did someone you were dating or going out with physically hurt you on purpose? (Count things such as being hit, slammed into something, or injured with an object or weapon.)
7. *Forced Sexual Activity or Dating Violence (past 12 months): Responded affirmatively to Q20 or Q22.
8. Bullied at School: Q23. During the past 12 months, have you ever been bullied on school property?
9. Bullied Electronically: Q24. During the past 12 months, have you ever been electronically bullied? (Count being bullied through texting, Instagram, Facebook, or other social media.).
10. *Bullied at School or Electronically: Endorsing either Q23 or Q24 as ‘yes.’
11. *Depression: Q25. During the past 12 months, did you ever feel so sad or hopeless almost every day for 2 weeks or more in a row that you stopped doing some usual activities?
12. *Suicidal Ideation: Q26. During the past 12 months, did you ever seriously consider attempting suicide?
13. Currently Smoke Cigarettes: QN32: During the past 30 days, on how many days did you smoke cigarettes?
14. Binge Drinking: QN42: During the past 30 days, on how many days did you have 4 or more drinks of alcohol in a row, that is, within a couple of hours (if you are female) or 5 or more drinks of alcohol in a row, that is, within a couple of hours (if you are male)?
15. *Marijuana Use: QN47: During the past 30 days, how many times did you use marijuana?
16. Marijuana Use-Lifetime:
17. Prescription Pain Medication Use-Lifetime: QN49: During your life, how many times have you taken prescription pain medicine without a doctor’s prescription or differently than how a doctor told you to use it?
18. Cocaine Use-Lifetime: QN50: During your life, how many times have you used any form of cocaine, including powder, crack, or freebase?
19. Inhalant Use-Lifetime: QN51: During your life, how many times have you sniffed glue, breathed the contents of aerosol spray cans, or inhaled any paints or sprays to get high?
20. Heroin Use-Lifetime: QN52: During your life, how many times have you used heroin (also called smack, junk, or China White)?
21. Methamphetamine Use-Lifetime: QN53: During your life, how many times have you used methamphetamines (also called speed, crystal meth, crank, ice, or meth)?
22. Ecstasy Use-Lifetime: QN54: During your life, how many times have you used ecstasy (also called MDMA or Molly)?
23. Injectable Drug Use-Lifetime: QN56: During your life, how many times have you used a needle to inject any illegal drug into your body?
24. *Used Illicit Drugs 3 or More Times: Reported using one or more of the following drugs 3 or more times during lifetime: (i) prescription pain medication with a prescription; (ii) cocaine; (iii) inhalants; (iv) heroin; (v) methamphetamines; (vi) ecstasy/MDMA; or (vii) used a needle to inject any illegal drug into your body.
25. Physically Active 5+ Days: QN78. During the past 7 days, on how many days were you physically active for a total of at least 60 min per day? (Add up all the time you spent in any kind of physical activity that increased your heart rate and made you breathe hard some of the time.) Endorsing 5 or more days was coded as ‘yes.’
26. *Physically Active 0 Days. QNPA0DAY: During the past 7 days, on how many days were you physically active for a total of at least 60 min per day? (Add up all the time you spent in any kind of physical activity that increased your heart rate and made you breathe hard some of the time.) Endorsing zero was coded as ‘yes.’
27. Playing on Sports Team: QN82: During the past 12 months, on how many sports teams did you play? (Count any teams run by your school or community groups.) Endorsing 1 or more was coded as “yes” for “Playing on Sports Team.”
28. *Insufficient Sleep: QN88. On an average school night, how many hours of sleep do you get? Those who endorsed 5 or fewer hours were coded as ‘yes.’
29. *Low Grades: QN89. During the past 12 months, how would you describe your grades in school? Those who endorsed ‘Mostly D’s’ or ‘Mostly F’s’ were classified as ‘yes.’

These questions were derived from the 2019 YRBS Data User’s Guide. Psychosocial Adversity Index: Sum of the following 11 variables marked with an asterisk (*).

### Statistical analyses

The percentages of students endorsing perceived cognitive impairment were stratified using demographic variables and other variables such as hours of sleep, academic grades, physical activity, depression, suicidality, adversity, and drug use. The proportions of students who endorsed experiencing cognitive difficulties were computed and compared using *χ*^2^ tests along several demographic and adversity-related variables. The proportions of subgroups endorsing cognitive impairment were stratified by gender, and an odds ratio (OR) was calculated for each analysis as an effect size. The OR was interpreted according to widely used criteria (i.e., ORs between 1.20 and 1.71 = small, ORs between 1.72 and 2.40 = medium, and ORs greater than 2.40 = large).

Binary logistic regressions were conducted separately for boys and girls using perceived cognitive impairment as the dependent variable. These analyses were conducted to determine which possible risk and adversity factors were significant predictors after accounting for depression and suicidality. Cognitive impairment was predicted by the following variables with the adjusted odds ratio (OR) reported for each analysis: (i) feeling unsafe or threatened at school, (ii) dating violence, (iii) sexual assault or abuse, (iv) being bullied, (v) depression, (vi) suicidal ideation, (vii) using marijuana, (viii) no physical activity, (ix) using illicit drugs, (x) insufficient sleep, and (xi) low grades. The specific wording of these questions is provided in Table 1. An OR above 1.00 with a 95% confidence interval (CI) not including 1.00 indicated that the predictor was associated with greater odds of endorsing the dependent variable, whereas an OR below 1.00 with a 95% CI not including 1.00 indicated that the predictor was associated with reduced odds of endorsing the dependent variable.

In addition, a “psychosocial adversity index” of convenience was created by summing positive endorsements to these same 11 questions relating to mental health, life adversity, substance use, and daily activity. The goal was to simply examine the practical issue of endorsing one or several questions, not to carefully model or quantify relative associations among these variables and the predictor. Those 11 questions are marked in both Table 1 and Table 3. Five subgroups were formed stratified by cumulative levels of adversity, based on the number of questions endorsed positively, as follows: 0 (none), 1, 2, 3, 4–5, and 6 or more. Gender differences were examined in those subgroups using *χ*^2^ tests. All statistical analyses were conducted using IBM SPSS Statistics 26.

**Table 2:** Percentages of high school students endorsing a significant problem with cognitive functioning.

Group	Total sample		Boys		Girls	
	*n*	%	*n*	%	*n*	%
Total sample	8,349	37.8	4,093	29.9	4,256	45.4
Age 14	936	39.4	411	29.4	525	47.2
Age 15	2,089	38.0	999	32.1	1,090	43.4
Age 16	2,245	39.0	1,090	29.4	1,155	48.1
Age 17	1,977	36.8	989	28.9	988	44.6
Age 18	1,102	35.6	604	29.3	498	43.2
Not Hispanic	6,072	36.8	2,985	29.1	3,087	44.2
Hispanic	2,191	40.3	1,051	31.5	1,140	48.5
Race						
White	4,117	36.7	2,007	28.4	2,110	44.6
Black	1,026	33.2	523	27.7	503	39.0
Hispanic/Latino	709	39.2	340	29.1	369	48.5
Asian	419	35.6	211	29.9	208	41.3
American Indian/Alaskan Native	70	50.0	43	46.5	27	55.6
Native Hawaiian	29	24.1	16	6.3	13	46.2
Multiple-Non-Hispanic	379	47.2	168	39.9	211	53.1
Multiple-Hispanic	1,482	40.9	711	32.6	771	48.5
Average hours of sleep on school night						
4 or Fewer Hours	822	59.2	418	50.2	404	68.6
5 h	1,259	47.3	556	37.1	703	55.3
6 h	2,117	38.7	960	30.9	1,157	45.2
7 h	2,286	32.0	1,161	25.5	1,125	38.8
8 h	1,384	26.3	726	19.8	658	33.4
9 h	307	28.0	176	19.3	131	39.7
10 or More Hours	103	45.6	57	43.9	46	47.8
Grades in School						
Mostly A’s	3,297	28.7	1,322	20.0	1,975	34.5
Mostly B’s	2,901	39.7	1,500	29.3	1,401	50.9
Mostly C’s	1,329	47.1	777	37.5	552	60.7
Mostly D’s	284	56.7	188	47.3	96	75.0
Mostly F’s	102	70.6	67	70.1	35	71.4
Participated on at least one sports team						
Yes	4,761	33.0	2,433	25.9	2,328	40.5
No	3,510	44.4	1,615	36.2	1,895	51.3
Physically active for 60+ Minutes 5+ Days						
Yes	3,770	30.9	2,200	25.7	1,570	38.2
No	2,544	43.6	1,856	35.1	2,658	49.5

These are the percentages of youth who said “yes” to the following question: “Because of a physical, mental, or emotional problem, do you have serious difficulty concentrating, remembering, or making decisions?” See Table 1 for the questions relating to sleep, grades, and physical activity.

**Table 3:** Percentages of high school students endorsing adversity, mental health problems, substance use, and having a significant problem with cognitive functioning.

Group	Total Sample		Boys		Girls	
	*n*	%	*n*	%	*n*	%
**Total sample**	8,349	37.8	4,093	29.9	4,256	45.4
*Low grades (Ds and Fs)	386	60.4	255	53.3	131	74.0
*Insufficient sleep (5 or fewer hours)	2,081	52.0	974	42.7	1,107	60.2
*No physical activity/exercise in past 7 days	1,341	46.3	534	38.4	807	51.5
**Adversity**						
*Felt Unsafe or Threatened at School	1,078	57.9	526	47.0	552	68.3
Felt Unsafe at School (and thus not gone to school)	646	61.0	265	48.3	381	69.8
Threatened or Injured with a Weapon at School	600	57.3	343	48.4	257	69.3
*Bullied Electronically or at School	2,063	56.3	758	47.0	1,305	61.7
Bullied Electronically	1,268	59.2	404	48.5	864	64.2
Bullied at School	1,633	57.6	616	47.9	1,017	63.5
*Ever Forced to have Sex (lifetime)	577	62.4	133	52.6	444	65.3
*Forced Sexual Activity or Dating Violence (past 12 months)	1,063	62.2	298	51.7	765	66.5
Forced Sexual Activity (past 12 months)	823	63.5	187	54.0	636	66.4
Physical Violence from Someone Dating (past 12 months)	422	62.3	169	50.9	253	70.0
**Depression/Suicidality**						
*Depression in Past Year	3,142	63.6	1,141	54.2	2,001	69.0
*Suicidal in Past Year	1,623	70.3	559	62.4	1,064	74.4
**Substance use**						
*Current Use of Marijuana (past month)	1,744	48.7	874	38.1	870	59.4
Current Binge Drinking (past month)	1,022	43.1	466	31.3	556	52.9
Currently Smoke Cigarettes	448	51.6	252	38.1	196	68.9
Marijuana (lifetime)	3,020	46.3	1,466	35.8	1,554	56.2
Prescription Pain Medication without Prescription (lifetime)	1,132	57.6	490	45.1	642	67.1
Cocaine (lifetime)	266	56.0	181	50.3	85	68.2
Inhalant (lifetime)	535	62.1	241	52.3	294	70.1
Heroin (lifetime)	96	54.2	75	50.7	21	66.7
Methamphetamines (lifetime)	128	53.1	90	46.7	38	68.4
Ecstasy (MDMA) (lifetime)	246	52.4	168	46.4	78	65.4
Illegal Injected Drug (lifetime)	87	52.9	63	49.2	24	62.5
*Used Illicit Drugs 3 or More Times (not including marijuana)	813	60.1	399	50.1	414	69.8

These are the percentages of youth, within subgroups, who said “yes” to the following question: “Because of physical, mental, or emotional problems, do you have serious difficulty concentrating, remembering, or making decisions?” See Table 1 for the questions and variable definitions. Psychosocial Adversity Index: Sum of the following 11 variables marked with an asterisk (*).

## Results

Data from 13,677 youth were included in the national database. There were 151 students who were missing data on their sex, and 72 who were missing data on their age. There were 13,442 students between the ages of 14 and 18 who did not have missing data on age or sex; and, of those, there were 8,349 who answered the question about their cognitive functioning. There was no difference in the sex distributions of those who answered the question compared to those who did not [*χ*^2^(1) =0.005, *p = 0.943*]. There was a small difference in age; those who answered the question were slightly older than those who did not [*t*(13,440) = −7.86, *p* < 0.001; Cohen’s *d* = 0.141]. Those who answered the question were more likely to self-identify as Hispanic (26.5%) than those who did not answer the question [16.0%; *χ*^2^(1) = 196.63, *p* < 0.001].

The final cohort included 8,349 students between the ages of 14 and 18 (*M* = 16.03 years, SD = 1.21). The sample included 4,093 boys (49.0%) and 4,256 girls (51%). The self-identified races and ethnicities of the cohort were as follows: 4,117 (49.3%) White students, 1,482 (17.8%) students who identified as multiple Hispanic ethnicities, 1,026 (12.3%) Black or African American students, 709 (8.5%) Hispanic/Latino student, 419 (5.0%) Asian students, 379 (4.5%) students who identified as multiple non-Hispanic ethnicities, 70 (0.8%) American Indian/Alaska Native students, and 29 (0.3%) Native Hawaiian/Other Pacific Island students.

### Proportions endorsing perceived cognitive impairment

A large minority of high school students reported serious difficulty concentrating, remembering, or making decisions as a result of having a physical, mental, or emotional problem (i.e., 37.8%). A greater percentage of girls (45.4%) than boys (29.9%) reported having cognitive problems [*χ*^2^(1) = 212.23, *p* < 0.001; OR = 1.95, 95% CI = 1.78–2.13]. Youth who reported being physically active for 60 min a day in at least 5 of the past 7 days, were less likely to endorse cognitive impairment (30.9%) compared to students who were not physically active (43.6%; *χ*^2^(1) = 141.19, *p* < 0.001; OR = 0.578, 95% CI = 0.528–0.633). Moreover, youth who participated on at least one sports team were less likely to endorse cognitive impairment (33.0%) than youth who did not play on a sports team (44.4%; *χ*^2^(1) = 110.49, *p* < 0.001; OR = 0.618, 95% CI = 0.565–0.676). Given that girls were more likely to endorse cognitive impairment than boys, the results are stratified by gender (Tables 2–5). High school students who reported experiencing adversity, mental health problems, and drug use were examined separately, in subgroups, to determine the percentages who endorsed having significant cognitive problems. These results are presented in Table 3.

**Table 4:** Logistic regressions predicting perceived cognitive impairment stratified by gender.

	Girls									
						95% CI			95% CI	
	*B*	*SE*	Wald	*p*	Adjusted OR	Lower	Upper	Unadjusted OR	Lower	Upper
Unsafe or Threatened at School	0.368	0.124	8.812	0.003	1.445	1.133	1.842	2.977	2.460	3.603
Bullied	0.363	0.087	17.371	<0.001	1.438	1.212	1.706	2.624	2.294	3.001
No Physical Activity	0.268	0.098	7.397	0.007	1.307	1.078	1.586	1.361	1.167	1.587
Low Grades	0.786	0.249	9.940	0.002	2.195	1.346	3.578	3.628	2.443	5.390
Insufficient Sleep	0.389	0.089	19.138	0.000	1.475	1.239	1.756	2.247	1.953	2.584
Sexual Abuse/Assault-Lifetime	−0.094	0.140	0.451	0.502	0.911	0.693	1.197	2.537	2.064	3.118
Forced Sex/Dating Violence-Year	0.236	0.113	4.376	0.036	1.266	1.015	1.579	2.849	2.417	3.358
Illicit Drugs 3+ Times	0.471	0.145	10.607	0.001	1.602	1.207	2.128	3.106	2.494	3.868
Currently Using Marijuana	0.193	0.098	3.890	0.049	1.212	1.001	1.468	2.047	1.758	2.382
Suicidality	0.665	0.100	44.023	<0.001	1.945	1.598	2.367	5.319	4.551	6.216
Depression	1.496	0.085	312.576	<0.001	4.462	3.780	5.266	7.011	6.121	8.030
	Boys									
						95% CI			95% CI	
	*B*	*SE*	Wald	*p*	Adjusted OR	Lower	Upper	Unadjusted OR	Lower	Upper
Unsafe or Threatened at School	0.271	0.126	4.630	0.031	1.311	1.024	1.678	2.344	1.945	2.824
Bullied	0.390	0.105	13.837	<0.001	1.476	1.202	1.813	2.542	2.161	2.991
No Physical Activity	0.235	0.121	3.801	0.051	1.265	0.999	1.603	1.545	1.279	1.867
Low Grades	0.829	0.159	27.314	<0.001	2.291	1.679	3.127	2.987	2.311	3.861
Insufficient Sleep	0.457	0.094	23.810	<0.001	1.580	1.315	1.899	2.139	1.840	2.486
Sexual Abuse/Assault-Lifetime	−0.021	0.245	0.007	0.933	0.979	0.606	1.584	2.751	1.944	3.895
Forced Sex/Dating Violence-Year	0.317	0.163	3.804	0.051	1.373	0.998	1.889	2.720	2.144	3.451
Illicit Drugs 3+ Times	0.271	0.137	3.938	0.047	1.312	1.003	1.715	2.617	2.123	3.227
Currently Using Marijuana	0.188	0.099	3.625	0.057	1.207	0.994	1.464	1.647	1.407	1.928
Suicidality	0.888	0.119	55.503	<0.001	2.430	1.924	3.069	5.096	4.224	6.149
Depression	0.983	0.093	110.761	<0.001	2.671	2.225	3.208	4.633	3.998	5.368

See Table 1 for definitions of these variables. CI = confidence interval and OR = odds ratio. For girls: *χ*^2^(11) = 1026.189, *p* < 0.001, Cox and Snell *R*^2^ = 0.240, Nagelkerke *R*^2^ = 0.321. For boys: *χ*^2^(11) = 569.158, *p* < 0.001, Cox and Snell *R*^2^ = 0.148, Nagelkerke *R*^2^ = 0.212.

**Table 5:** Comparing percentages of girls and boys with cognitive impairment stratified by the number of adversity items endorsed.

Adversity questions endorsed		Boys	Girls				
	*n*	% Yes	% Yes	*χ* ^2^	*p*	Odds Ratio	95% CI
0 (none)	2,232	12.8	15.3	2.836	0.092	1.230	0.966–1.565
1	2,075	24.1	31.5	13.906	<0.001	1.443	1.190–1.751
2	1,478	33.7	44.8	19.046	<0.001	1.596	1.293–1.969
3	1,020	49.3	63.5	20.396	<0.001	1.787	1.388–2.301
4–5	1,097	60.9	72.5	15.439	<0.001	1.687	1.298–2.193
6 or More	447	73.0	83.2	5.909	0.015	1.827	1.119–2.983

*n* = sample size; % = percentage; *χ*^2^ = Chi square; *p* = probability value; CI = confidence interval. The 11 questions comprising the psychosocial adversity index are marked with an asterisk in Tables 1, 3.

### Independent predictors of perceived cognitive impairment

Binary logistic regression was used to examine the associations between perceived cognitive impairment and adversity, mental health, and lifestyle variables. These analyses were conducted separately by gender. The unadjusted and adjusted results are presented in Table 4. In the unadjusted analyses, strong predictors for girls included depression, suicidality, low grades, using illicit drugs, feeling unsafe or threatened at school, having forced sex or being subjected to dating violence in the past year, being bullied, and lifetime sexual abuse—with all ORs greater than 2.5. In the unadjusted analyses, strong predictors for boys included suicidality, depression, low grades, lifetime sexual abuse, having forced sex or being subjected to dating violence in the past year, using illicit drugs, and being bullied—with all ORs greater than 2.5.

The multivariable logistic regression model for predicting perceived cognitive impairment in girls was significant, *χ*^2^(11) = 1,026.189, *p* < 0.001. Approximately 32% of the variance (Nagelkerke *R*^2^) in perceived cognitive impairment was explained by the set of predictors. Significant independent predictors of perceived cognitive impairment for girls included depression, suicidality, getting very low grades, a lifetime history of using illicit drugs, insufficient sleep, feeling unsafe or being threatened at school, being bullied, no physical activity or exercise in the past week, having forced sex or being subjected to dating violence in the past year, and current marijuana use.

The multivariable logistic regression model for predicting perceived cognitive impairment in boys was significant, *χ*^2^(11) = 569.158, *p* < 0.001, and 21% of the variance (Nagelkerke *R*^2^) in perceived cognitive impairment was explained by the set of predictors. Significant independent predictors of perceived cognitive impairment for boys included depression, suicidality, obtaining very low grades, insufficient sleep, being bullied, having forced sex or being subjected to dating violence in the past year, feeling unsafe or being threatened at school, and a lifetime history of using illicit drugs.

### Psychosocial adversity index

**Figure 1:**
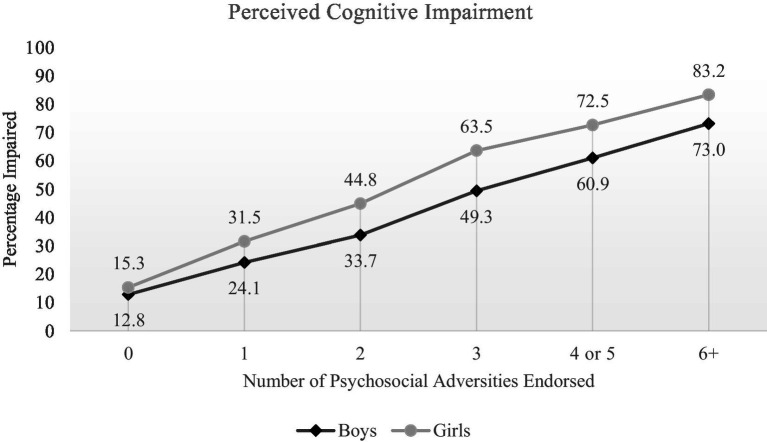
Percentages endorsing perceived cognitive impairment stratified by number of psychosocial adversities experienced. The 11 questions comprising the psychosocial adversity index are marked with an asterisk in Tables 1, 3.

A psychosocial adversity index was created by summing positive endorsements to 11 specific adversity items. Those 11 items are marked with an asterisk in Table 3. For girls, the median number of items endorsed was 2 (IQR = 1–4; range = 0–15). For boys, the median number of items endorsed was 1 (IQR = 0–3; range = 0–13). Girls endorsed a greater number of the adversity variables than the boys (Mann Whitney U = 10,319,102.50, *p* < 0.001). The percentages of boys and girls, stratified by their score on the psychosocial adversity index, who endorsed cognitive impairment are presented in Figure 1. As seen in Table 5 and Figure 1, for adolescents who reported no psychosocial adversity, only a small percentage endorsed perceived cognitive impairment, and there were no significant difference in percentages between girls and boys. There was a strong linear association between the number of adversities endorsed and the percentage endorsing cognitive impairment, in both girls and boys, and girls experiencing greater adversity were significantly more likely to endorse cognitive impairment than boys with similar levels of adversity.

## Discussion

This study revealed a remarkably high rate of perceived cognitive impairment amongst high school students in the United States (i.e., 30% of boys and 45% of girls). Depression and suicidality were strongly, and independently, related to perceived cognitive impairment. This, of course, was expected given that perceived difficulties with cognitive functioning are a cardinal symptom of depression (American Psychiatric Association, 2013), and the question itself referred to having cognitive difficulties ‘because of a physical, mental, or emotional problem.’ The survey did not include questions about a wide range of developmental and health conditions that might be associated with perceived cognitive impairment such as ADHD, learning disorders, autism spectrum disorders, anxiety disorders, general medical problems that could affect brain health, moderate or severe traumatic brain injury, brain tumors, or epilepsy. Therefore, the associations between many possible health conditions and perceived cognitive impairment are unknown.

Regular physical activity, and participating in sports, were associated with a decreased likelihood of endorsing cognitive impairment in the present study. Researchers have reported that greater physical activity in adolescents is associated with better cognitive functioning, both subjectively and on neuropsychological testing (Dewald-Kaufmann et al., 2013). Researchers have also reported that participation in team sports, and a high level of physical activity, are associated with better self-esteem and greater life satisfaction, and lower risk for psychological distress (Steptoe and Butler, 1996; Eime et al., 2013; McMahon et al., 2017; Guddal et al., 2019). Adolescents who are physically active, and participate in sports, are at lower risk for experiencing depression (Babiss and Gangwisch, 2009; He et al., 2018) and suicidality (Sabo et al., 2005; Brown et al., 2007; Taliaferro et al., 2008; Sibold et al., 2015).

Reporting very poor grades was independently related to perceived cognitive impairment (Table 4). There could be a bidirectional relationship between perceived cognitive impairment and very poor grades, in that low grades could be a consequence of having cognitive problems, cognitive impairment could be related to psychological distress associated with poor grades, or both. Insufficient sleep also was independently related to perceived cognitive impairment (Table 4). This is consistent with prior studies of high school student athletes reporting that insufficient sleep is associated with greater physical, emotional, and cognitive symptoms during baseline preseason health evaluations (McClure et al., 2014; Silverberg et al., 2016; Terry et al., 2021; Moran and Ingargiola, 2022), psychosocial adversities, such as being bullied, having forced sex or being subjected to dating violence in the past year, and feeling unsafe or being threatened at school, were independently associated with perceived cognitive impairment in both boys and girls—even after controlling for the associations with depression and suicidality (Table 4). A lifetime history of using illicit drugs three or more times also was an independent predictor after controlling for depression and suicidality.

We created a psychosocial adversity index derived by summing positive responses to 11 variables assessing different aspects of mental health problems, substance use, bullying, sexual abuse, low grades, and health behaviors (Tables 1, 3). More than 25% of students endorsed none of these 11 variables, and for those students the proportions endorsing perceived cognitive impairment were low and there was no significant difference between girls and boys (Table 5, Figure 1). In contrast, there was a clear linear association between the number of psychosocial stressors endorsed and the proportions of girls and boys endorsing perceived cognitive impairment (Figure 1), with significant gender differences present at all levels (Table 5).

### Limitations

There are several limitations in this study relating to the nature of the Youth Risk Behavior Survey. The survey was administered to students at school during a class period in which respondents were surrounded by their peers. The self-report nature of the survey and the circumstances surrounding its administration could lead to a number of biases in student responses. The CDC implemented a system check that attempted to identify surveys that reflected mischievous responding. However, there are other possible response biases that could have impacted the responding of some students such as social desirability, under reporting, over-claiming/over-reporting, extreme response styles, acquiescence response styles, yea-saying, or nay-saying. Attempting to study different types of response styles and response biases is beyond the scope of this study.

The methodology of this study was a cross-sectional survey, which does not allow us to draw causal inferences. Students were not asked any further questions about the scope of their cognitive impairment or about the duration of the impairment. We examined a large number of demographic and psychosocial adversity variables. We did not attempt to model perceived cognitive impairment among students; but rather we aimed to examine the association of cognitive impairment with these demographic and psychosocial adversity variables—especially after controlling for depression and suicidality because these two variables are related to the question about perceived cognitive impairment. Due to the large number of variables included in our analyses, it is expected that there is some overfitting of the statistical model; however, this is mitigated by the very large sample size.

## Conclusion

A remarkably large proportion of high school students in the United States reported experiencing serious difficulty with their cognitive functioning over the past year. Girls were significantly more likely to endorse perceived cognitive difficulties compared to boys. There was a strong association between perceived cognitive impairment and the experience of psychosocial adversity. However, among students who do not report experiencing psychosocial adversity, there was no significant gender difference in the percentages endorsing perceived cognitive difficulties and, overall, these students reported relatively low rates of perceived cognitive impairment compared to students who endorsed experiencing psychosocial adversity. Future research might better define and understand perceived cognitive impairment in high school students, its underlying causes and functional correlates, the extent to which it is related to social determinants of health, and how to promote cognitive self-efficacy and improved functioning.

## Data availability statement

The datasets presented in this study can be found in online repositories. The names of the repository/repositories and accession number(s) can be found at: https://www.cdc.gov/healthyyouth/data/yrbs/. The survey data from 1991 through 2019 are publicly available (https://www.cdc.gov/healthyyouth/data/yrbs/). The survey questions, recall periods, response options, and definitions of each variable are provided in the 2019 YRBS questionnaire and data user’s guide available on the website (https://www.cdc.gov/healthyyouth/data/yrbs/).

## Ethics statements

The Institutional Review Board of the CDC approved the protocol for the YRBS. Survey procedures were designed to protect students’ privacy by allowing for anonymous and voluntary participation. Before survey administration, local parental permission procedures were followed.

## Author contributions

GI conceptualized the study, conducted the literature review, conceptualized and conducted the statistical analyses, wrote portions of the manuscript, and agrees to be accountable for the content of the work. II assisted with reviewing literature, completed tables and figures, wrote portions of the manuscript (i.e., abstract, introduction, methods, and discussion), and agrees to be accountable for the content of the work.

## Funding

Grant Iverson, Ph.D. acknowledges unrestricted philanthropic support from ImPACT Applications, Inc., the Mooney-Reed Charitable Foundation, and the Spaulding Research Institute. These entities were not involved in the study design, collection, analysis, interpretation of data, the writing of this article or the decision to submit it for publication. The authors declare no other funding sources or competing interests.

## Conflict of interest

The authors declare that the research was conducted in the absence of any commercial or financial relationships that could be construed as a potential conflict of interest.

## Publisher’s note

All claims expressed in this article are solely those of the authors and do not necessarily represent those of their affiliated organizations, or those of the publisher, the editors and the reviewers. Any product that may be evaluated in this article, or claim that may be made by its manufacturer, is not guaranteed or endorsed by the publisher.

## Footnotes

^1^ https://www.cdc.gov/healthyyouth/data/yrbs/
